# Education in *Haor* areas of Bangladesh: A mixed-methods study on academic performance, school attendance, and school intervention

**DOI:** 10.1371/journal.pone.0348890

**Published:** 2026-05-11

**Authors:** Md. Ismail Hossain, Iftakhar Ahmad

**Affiliations:** 1 Department of Social Work, Shahjalal University of Science and Technology, Sylhet, Bangladesh; 2 Department of Social Work, Shahjalal University of Science and Technology, Sylhet, Bangladesh; West University of Timisoara: Universitatea de Vest din Timisoara, ROMANIA

## Abstract

This study examines the factors affecting secondary education in the Tanguar *Haor* region of Bangladesh, employing a sequential explanatory design. Quantitative data were collected from 189 students across 10 randomly selected secondary schools using structured questionnaires, while qualitative insights were obtained through semi-structured interviews with 20 teachers and guardians, guided by an interview guideline. Descriptive and inferential analyses, including chi-square and logistic regression, were applied to quantitative data, and thematic analysis was used for qualitative data. Data analysis revealed that academic performance was strongly influenced by the number of teachers, bullying, peer support, class level, family income, teachers’ behavior, learning difficulty, marital status, and smartphone usage; school attendance was associated with parental education, school distance, and peer influence, while absenteeism was affected by similar academic, social, and household factors. Logistic regression indicated that students from higher-income families were more likely to achieve good academic outcomes (B = 2.129, p < .001, OR = 8.65, 95% CI [3.15, 23.72]), and peer support positively influenced performance (B = 1.359, p = .006, OR = 4.02, 95% CI [1.42, 10.63]). School attendance was positively predicted by parental education (B = 0.910, p = .009, OR = 2.48, 95% CI [1.25, 4.93]) and negatively influenced by greater school distance (B = –0.819, p = .013, OR = 0.44, 95% CI [0.23, 0.84]) and limited peer help (B = –0.832, p = .037, OR = 0.44, 95% CI [0.20, 0.95]). Chronic absenteeism increased with bullying (B = 1.361, p < .001, OR = 3.90, 95% CI [1.90, 7.99]) and punitive teacher behavior (B = 1.146, p = .009, OR = 3.15, 95% CI [1.33, 7.43]) and decreased with household work (B = –1.082, p = .022, OR = 0.34, 95% CI [0.14, 0.85]), higher family income (B = –1.350, p < .001, OR = 0.26, 95% CI [0.13, 0.53]), and peer influence (B = –0.920, p = .013, OR = 0.40, 95% CI [0.19, 0.82]). Qualitative findings corroborated these results, highlighting socio-economic and community-level barriers. Despite applying school intervention, educational challenges persist in *Haor* areas. Finally, the study recommends context-specific interventions to address challenges and enhance educational outcomes in this ecologically vulnerable region.

## Introduction

The formal education system in Bangladesh comprises five levels: pre-primary, primary, secondary, higher secondary, and tertiary education. Primary education (Grades 1–5) is administered by the Ministry of Primary and Mass Education (MoPME), while secondary and higher secondary education (Grades 6–12) are overseen by the Ministry of Education through the Directorate of Secondary and Higher Education (DSHE) [1]. Over the past decades, the country has made considerable progress in expanding access to schooling. However, persistent challenges such as irregular attendance and student absenteeism, particularly at the secondary level, continue to constrain educational outcomes. In education research, school absenteeism and attendance problems are often conceptualized as interconnected phenomena. For example, Kearney et al. [2] used “school attendance problems and absenteeism” as closely related constructs within a broader framework of school participation difficulties. In this context, academic performance generally refers to the extent to which students achieve educational goals [3].

Attendance difficulties and poor academic outcomes are particularly prevalent in rural and remote schools, where structural disadvantages persist. These include shortages of qualified teachers, outdated instructional materials, and limited co-curricular opportunities that affect students’ learning experiences and engagement [4]. In many rural communities, children are also involved in household responsibilities or income-generating activities, especially during peak agricultural seasons, which often reduces their school attendance and academic engagement [5]. Gender-specific constraints further affect educational participation, as girls may face early marriage, domestic responsibilities, and restricted mobility that hinder their continued schooling [6]. Such barriers are compounded by limited public investment in education, restricting the availability of resources for schools in disadvantaged areas [7].

Broader socioeconomic and environmental conditions also shape educational performance and participation. Poverty, child labor, parental illiteracy, and early marriage interact with environmental challenges such as seasonal flooding, poor transportation, and inadequate school infrastructure, collectively impeding consistent school attendance and academic achievement among marginalized populations [8]. Additionally, the persistence of corporal punishment in some schools, despite policy discouragement, has been linked to poor attendance, lower academic performance, increased dropout risk, and psychological distress among students [9]. To address these challenges, various school-level interventions have been implemented, including teacher training programs, improvements in school climate, and community-based support mechanisms. Nevertheless, empirical evidence on the effectiveness of these initiatives in improving attendance and learning outcomes remains limited [10].

Research on rural education in Bangladesh has largely focused on broad systemic challenges [11], while the distinctive barriers faced by students in *Haor* regions remain relatively underexplored. *Haor* areas are characterized by conditions that often intensify educational inequalities. Although some studies have examined the quality of government secondary education within specific divisional contexts [12], there is a notable scarcity of empirical research addressing the educational experiences of students in these flood-prone regions. Previous investigations have identified environmental disruptions as important factors affecting school attendance and learning [13]. However, few studies have systematically examined how contextual challenges interact with social and institutional factors to shape students’ academic performance and attendance patterns. Furthermore, while recent studies have examined the educational consequences of the pandemic in marginalized wetland communities [14], they also overlook the complex local realities that influence student learning and attendance in *Haor* regions.

Addressing this gap, the present study adopts a mixed-methods design, combining quantitative data from secondary-level students with qualitative data from school administrators, teachers, and parents. By integrating quantitative and qualitative approaches, the study aims to provide both empirical breadth and contextual depth in understanding the factors associated with academic performance, attendance, and absenteeism among students in *Haor* areas. The findings are expected to yield locally grounded insights that can inform policies and interventions to strengthen equitable access to education. In doing so, the study contributes to Bangladesh’s efforts to achieve United Nations Sustainable Development Goal 4, which seeks to ensure inclusive and equitable quality education for all [15].

## Profile of the *Haor* Community

The *Haor* region of northeastern Bangladesh represents one of the country’s most socioeconomically and environmentally vulnerable areas. This wetland ecosystem is characterized by vast seasonal water bodies and low-lying floodplains that remain submerged for several months each year. Recurrent flash floods during the monsoon season frequently inundate villages, disrupt transportation networks, and damage local infrastructure, limiting access to essential services [16]. These environmental conditions create substantial barriers to social and economic stability, with direct implications for educational access and continuity. Socioeconomic vulnerability further intensifies these challenges. A household survey covering 2,340 families in the *Haor* region reported that approximately 25% of households live below the lower poverty line and an additional 55% fall below the upper poverty line, indicating widespread economic hardship [17]. Basic services such as sanitation, safe drinking water, and electricity remain limited in many disadvantaged communities, further reinforcing structural inequalities. Such conditions shape household priorities and often reduce families’ ability to support children’s schooling, particularly when livelihoods depend on seasonal agricultural or fishing activities [16].

Educational infrastructure and resources in the *Haor* region also face significant constraints. Long distances between villages and schools, limited transportation facilities, insufficient teacher training, and high student–teacher ratios often undermine the quality of education in rural schools. Classrooms are frequently overcrowded, and learning materials are limited, further affecting teaching and learning conditions [11,12]. These challenges are reflected in regional education indicators: literacy rates remain around 38%, net enrollment is approximately 82%, and primary school dropout rates reach 44%, exceeding the national average of 40%. Attendance rates are also relatively low, with about 74% in primary education and only 37% in secondary education, while approximately 60% of students transition from primary to secondary school [1]. Environmental disruptions and socioeconomic constraints together shape students’ educational participation and academic performance. Flooding often interrupts school operations and damages transportation routes, making regular attendance difficult. During the monsoon season, many students travel to school by boat, which introduces safety risks and additional financial burdens for families [14]. Seasonal labor demands in agriculture and fishing further reduce students’ availability for schooling, while child labor and household responsibilities contribute to irregular attendance and disengagement from learning activities [5]. The environmental and socioeconomic challenges make this region a critical setting for examining how these contextual factors affect educational achievement.

## Objectives of the Study

The general objective of the study is to explore the causes of poor educational outcomes and potential initiatives to address them, with a view to improving secondary education in *Haor* areas of Bangladesh. However, the specific objectives are:

i To explore the factors influencing the educational performance of *Haor* children in Bangladesh;
ii To examine factors associated with irregular attendance among *Haor* students;
iii To find out the initiatives being taken by the school authorities to resolve educational challenges, and
iv To identify potential school- and community-based initiatives to address attendance and learning challenges faced by *Haor* students.

## Methodology of the study

### Research design

This study employed a sequential explanatory design to comprehensively examine the factors influencing secondary education in the Tanguar *Haor* region of Sunamganj District, Bangladesh. This region is an ecologically sensitive, socioeconomically vulnerable wetland area located in the Dharmapasha and Tahirpur Upazilas of Sunamganj District. This approach was chosen to capture both measurable patterns of academic performance and school attendance-related student behaviors, as well as the contextual and lived experiences of stakeholders.

### Sample and sampling

The study population comprised all secondary-level students enrolled in schools in the Tanguar *Haor* region in Sunamganj District, Bangladesh. To obtain a representative sample, a multistage random sampling strategy was employed. In the first stage, 10 secondary schools were randomly selected from across the Tanguar *Haor* region. In the second stage, within each selected school, students were initially screened by teachers to identify those demonstrating inattentiveness, irregular attendance, and absenteeism. Teachers were considered an appropriate source for identifying such students because of their familiarity with students’ academic behaviors.

From the screened list of students, twenty students were randomly selected from each school, ensuring representation from all grade levels. Specifically, four students were selected from each grade from class six to class ten, resulting in an intended quantitative sample of 200 students. During data collection, eleven students from the last two schools were unavailable, resulting in a final sample of 189 students. Although students were selected from multiple schools, the analysis focused on overall patterns of academic performance, school attendance, and absenteeism among students in the *Haor* context rather than on school-level comparisons.

In addition to the student survey, qualitative data were collected from key informants to complement and contextualize the quantitative findings. These participants included five members of school management committees (one from each of the first five selected schools), five educated guardians, and the head teacher of each selected school. These informants were purposively selected for their direct involvement in school management and for their experience with students’ educational pursuits. Their perspectives provided insights into existing school initiatives and potential interventions to address attendance, learning difficulties, and absenteeism in the *Haor* context.

The adequacy of the quantitative sample size was assessed in relation to the statistical analyses employed in this study. Logistic regression models were used to identify predictors of academic performance, school attendance, and absenteeism. The sufficiency of the sample was evaluated using the events-per-variable (EPV) criterion, which recommends at least 10 events per predictor variable to ensure stable parameter estimates. In the present study, the academic performance model had an EPV of 14.67, the school attendance model had an EPV of 12.00, and the absenteeism model had an EPV of 10.14. These values meet or exceed the recommended threshold, indicating that the sample size is adequate for reliable logistic regression analysis. Similar recommendations regarding EPV thresholds for logistic regression stability have been documented in methodological research [18].

### Key variables

This study employed a set of contextually relevant independent and dependent variables to examine students’ educational engagement and academic performance. All variables were carefully operationalized to reflect the lived realities of the study population and were measured using self-reported data from students. Three key outcome variables were analyzed: ‘academic performance’, ‘school attendance’, and ‘absenteeism’. ‘Academic performance’ reflects students’ achievement, as assessed by their most recent annual examination results. It is measured using students’ most recent annual examination scores, with scores below 50% categorized as poor performance and scores of 50% or above as satisfactory performance. We used the term irregularity in the context of ‘school attendance’ to refer to the consistency of attendance and broader patterns of student engagement in academic activities, including late arrival, early departure, partial attendance, truancy, and missing classes during the rainy season. In this study, ‘absenteeism’ is classified into two distinct forms based on the number of consecutive school days missed without justification: occasional absenteeism (fewer than 5 consecutive days) and chronic absenteeism (5 or more consecutive days).

The independent variables measured in this study include ‘household work’, ‘income-earning activities’, ‘parental education’, ‘family income’, ‘distance from school’, ‘smartphone use’, ‘bullying’, ‘peer help for study’, and ‘teachers’ behavior’. ‘Household work’ refers to the domestic responsibilities students regularly undertake, such as household chores. ‘Income-earning activities’ capture whether the student is involved in any form of paid work that contributes to household income. ‘Parental education’ is defined as the level of education attained by either parent. ‘Family income’ is measured by the student’s perception of whether household income is adequate to cover family expenditures. ‘School distance’ is defined by proximity: schools more than 2 kilometers from the student’s home are considered far, while those within 2 kilometers are considered nearby. ‘Smartphone use’ is assessed by whether students regularly use smartphones for entertainment. ‘Bullying’ is examined to determine whether students experience verbal or physical harassment while going to or returning from school. ‘Peer help for study’ means academic support received from classmates. Finally, ‘teachers’ behavior’ is determined by students’ perceptions, with some being supportive and others punitive. The coding systems for both dependent and independent variables are shown in Table 1.

**Table 1:** Key variables and their coding system.

Variable names	Coding system
***Dependent variables***	
Academic performance	Poor = 0, Good = 1
School attendance	Irregular = 0, Regular = 1
Absenteeism	Occasional = 0, Chronic = 1
***Independent variables***	
Doing household work	No = 0, Yes = 1
Income-earning activities	No = 0, Yes = 1
Parental education	Below secondary = 1, Secondary = 2, Above secondary = 3
Family income	Inadequate = 0, Adequate = 1
School distance	No (nearby) = 0, Yes (far) = 1
Smartphone usage	No = 0, Yes = 1
Bullying	No = 0, Yes = 1
Peer help for study	No = 0, Yes = 1
Teachers’ behavior	Punitive = 0, Supportive = 1

### Instruments development

A structured questionnaire was developed to collect quantitative data from students. To explore contextual challenges, the questionnaire was prepared with consideration of context-specific realities. The development process included an extensive literature review to identify relevant thematic areas, including socio-economic aspects, environmental issues, disciplinary practices, academic performance, parental expectations, peer relationships, irregularity, and absenteeism. During the pretest of this instrument, consultations were made with local teachers and education officials to ensure cultural and contextual relevance. Pre-testing informed revisions in language and clarity, as well as the inclusion of new questions. The questionnaire directly addressed the study’s first three objectives. The fourth objective was also partially covered by the questionnaire.

Furthermore, qualitative data were collected following a semi-structured interview guide. This instrument addressed the objectives related to school initiatives and the potential interventions to address the challenges found in this study. The instrument included open-ended questions focusing on existing interventions by school authorities to address irregularity, absenteeism, and performance issues. Questions were designed based on a review of relevant literature and the researchers’ prior experience in researching related topics in rural contexts.

### Data collection

To facilitate data collection, four trained data collectors with prior fieldwork experience and familiarity with mixed-methods research were recruited. They received a comprehensive orientation on the study objectives, research instruments, and interview protocols to ensure methodological consistency and ethical engagement with participants. Participant recruitment was conducted during the latter half of February 2025 through home and school visits, with assistance from school teachers. The quantitative data collection phase took place between 03 March and 25 April 2025, followed by qualitative interviews that continued until 10 May 2025. Although the research instruments were initially developed in English, all interviews were conducted in Bangla, the national language of Bangladesh, to create a linguistically and culturally comfortable environment for the students, enabling more spontaneous and authentic responses. Each student interview lasted approximately 60 minutes and was conducted at the student’s home during off days. Key informant interviews (KIIs) with teachers were conducted at their respective offices during regular working days, scheduled at times when they were free. KIIs with guardians were conducted in their homes. Interviews with teachers typically lasted around 40 minutes, while KIIs with guardians lasted over an hour. Qualitative responses were recorded in notebooks and simultaneously captured through mobile audio recording to ensure accuracy.

### Data analysis

Quantitative data analysis was conducted using the Statistical Package for the Social Sciences (SPSS). During the qualitative interviews, quantitative data were entered into SPSS, and preliminary analyses were conducted between 16 and 30 May 2025. Then, an analysis was conducted to identify key issues and patterns related to student performance, school irregularity, and absenteeism. Descriptive statistics were used to summarize the variables, while inferential analyses examined their associations. Chi-Square tests were employed to examine relationships between dependent variables and socio-demographic variables. Moreover, binary logistic regression models were used to identify predictors of academic performance, school attendance, and absenteeism. To complement and deepen the interpretation of quantitative findings, qualitative data were analyzed thematically. All interviews were transcribed, edited, and coded before theme identification. Qualitative data analysis explored contextual insights into the lived experiences of *Haor* people, the challenges in *Haor* children’s education, the school environment, and perspectives on potential interventions. These findings were used to support and enrich the quantitative results, offering a more nuanced understanding of the educational issues in *Haor* areas. An integrative analysis combining quantitative and qualitative findings was undertaken throughout June 2025.

### Ethical considerations

Ethical standards were rigorously maintained throughout the study. All participants provided informed consent after being briefed on the study’s objectives, procedures, voluntary nature of participation, and confidentiality assurances. For students, consent was obtained from their guardians, in addition to the students’ own assent. Consent was documented in writing for both students and guardians. Special attention was given when discussing sensitive topics to ensure participants’ comfort and understanding. Anonymity was maintained by removing personal identifiers from transcripts, and all data were handled with strict confidentiality. This study was approved by the Shahjalal University of Science and Technology Research Ethics Board (SREB), Sylhet-3114, Bangladesh.

## Findings of the study

### Factors influencing academic performance

#### Punitive practices, familial responsibilities, and economic factors.

A combination of individual, familial, and school-level factors influenced academic performance. Data suggest that 72.2% of male and 66.7% of female students demonstrated good performance, with students aged 13–16 years performing best. Classes Seven (94.4%) and Ten (82.5%) achieved higher performance, whereas Class Nine had the lowest performance (47.5%). Difficulty in understanding lessons was associated with poor outcomes. Students from higher-income families performed better (86.7%), and parental education above secondary level was associated with higher achievement (72.7%). Peer support improved outcomes (81.9%), whereas bullying (46.3%) and punitive teacher behavior (60.3%) reduced performance (see Table 2).

**Table 2:** Distribution of academic performance, school attendance, and absenteeism by selected variables.

Variables	Categories	Academic Performance		School Attendance		Absenteeism	
		Poor (Row %)	Good (Row %)	Irregular (Row %)	Regular (Row %)	Occasional (Row %)	Chronic (Row %)
Sex	Male	27.8	72.2	47.2	52.8	66.3	33.7
	Female	33.3	66.6	37.0	63.0	56.8	43.2
Age (years)	11–12	37.1	62.9	34.3	65.7	54.3	45.7
	13–14	26.5	73.5	41.2	58.8	64.7	35.3
	15–16	27.7	72.3	47.7	52.3	64.6	35.4
	17–18	38.1	61.9	47.6	52.4	61.9	38.1
Class	Six	31.4	68.6	37.1	62.9	62.9	37.1
	Seven	5.6	94.4	36.1	63.9	88.9	11.1
	Eight	42.1	57.9	36.8	63.2	50.0	50.0
	Nine	52.5	47.5	62.5	37.5	42.5	57.5
	Ten	17.5	82.5	40.0	60.0	70.0	30.0
Number of teachers	6–10	55.6	44.4	45.6	54.4	41.1	58.9
	11–15	7.3	92.7	41.2	58.8	83.5	16.5
	16–20	7.9	92.1	35.7	64.3	71.4	28.6
Difficulty in understanding lessons	Yes	50.0	50.0	45.7	54.3	38.6	61.4
	No	18.5	81.5	41.2	58.8	76.5	23.5
Family income	Yes	13.3	86.7	48.9	51.1	77.8	22.2
	No	45.5	54.5	37.4	62.6	48.5	51.5
Parental education	Below secondary	32.2	67.8	48.50	51.50	59.7	40.3
	Secondary	25.6	74.4	27.9	72.1	67.4	32.6
	Above secondary	27.3	72.7	31.5	68.5	68.2	31.8
School distance	Yes	32.9	67.1	52.9	47.1	61.2	38.8
	No	27.9	72.1	34.6	65.4	63.5	36.5
Smartphone usage	Yes	21.1	78.9	42.2	57.8	70.3	29.7
	No	49.2	50.8	44.3	55.7	45.9	54.1
Bullying	Yes	53.7	46.3	37.8	62.2	43.9	56.1
	No	12.1	87.9	46.7	53.3	76.6	23.4
Peer help for study	Yes	18.1	81.9	48.0	52.0	72.4	27.6
	No	54.8	45.2	32.3	67.7	41.9	58.1
Teachers’ behavior	Supportive	39.7	60.3	46.3	53.7	55.1	44.9
	Punitive	5.7	94.3	34.0	66.0	81.1	18.9

Teachers’ misbehaviors remain highly prevalent in the studied schools. Threats of punishment were reported by 90.5% of students, and scolding by 54.0%, often resulting in profound sadness (42.3%) and fear (42.3%). Half of the students indicated disinterest in attending school the following day after experiencing such incidents. Physical punishments were common in *Haor* areas. Hitting with objects was most common (90%), followed by twisting ears (66.7%) and public humiliation (62%). Other frequent punishments included forced standing (58%) and kneeling (46%), while pulling ears, extra tasks, and stretching hair affected 43.3%, 41%, and 29.2% of students, respectively. These measures were typically employed in response to irregular attendance (86.8%), non-compliance (63%), late arrival (61%), and inattentiveness (59.3%), with academic and material-related issues also significant.

Qualitative data reinforce these findings, revealing that corporal punishment remains a routine disciplinary approach in many schools, ultimately diminishing students’ interest in learning. One guardian shared the following statement:

*A few days ago, my son went to school fully prepared for his lesson. When the teacher started taking the performance from each one angrily and beating them for failing to give the lesson properly, he got scared and could not respond correctly. Then the teacher smote him indiscriminately. Since then, he has lost interest in preparing his lessons, as he believes he would be beaten regardless of his performance.*

One school management personnel’s statement illustrates the pervasive impact of punitive teacher practices on student engagement:

*Even if a student does their homework perfectly, the teacher sometimes scolds or hits them if their response to the teacher’s query is slow. Many children now fear engaging in classroom discussions.*

These qualitative observations are consistent with the quantitative results, which indicate that punitive teacher behavior significantly reduces academic performance (*B* = –2.740, *p* < .001, *OR* = 0.065, 95% *CI* [0.015, 0.281]). Logistic regression analysis identified additional significant predictors of academic performance. Students engaged in household work were more likely to achieve good academic performance (*B* = 1.008, *p* = .041, *OR* = 2.74, 95% *CI* [1.041, 7.215]). Family income emerged as the strongest positive predictor (*B* = 2.129, *p* < .001, *OR* = 8.41, 95% *CI* [2.998, 23.578]). Peer support for studies was also a significant positive factor (*B* = 1.359, *p* = .008, *OR* = 3.89, 95% *CI* [1.424, 10.632]), whereas experiences of bullying negatively affected academic outcomes (*B* = –2.529, *p* < .001, *OR* = 0.08, 95% *CI* [0.029, 0.220]) (see Table 3).

**Table 3:** Logistic regression analysis predicting academic performance.

Variables	Academic Performance							
	B	SE	Wald	df	*P*-value	OR (Exp(B))	95% CI for OR	
							Lower	Upper
Doing household work	1.008	0.494	4.170	1	0.041	2.741	1.041	7.215
Income-earning activities	−2.218	1.320	2.821	1	0.093	0.109	0.008	1.448
Parental education	0.290	0.478	0.370	1	0.543	1.337	0.524	3.409
Family income	2.129	0.526	16.377	1	<0.001	8.408	2.998	23.578
School distance	−0.307	0.478	0.413	1	0.521	0.736	0.289	1.876
Smartphone usage	0.668	0.526	1.615	1	0.204	1.951	0.696	5.472
Bullying	−2.529	0.519	23.785	1	<0.001	0.080	0.029	0.220
Peer help for study	1.359	0.513	7.018	1	0.008	3.891	1.424	10.632
Teachers’ behavior	−2.740	0.750	13.348	1	<0.001	0.065	0.015	0.281
Constant	2.062	0.865	5.676	1	0.017	7.859	—	—

[*Note. N = 189.P <.05 values are significant. OR = Odds Ratio; CI = Confidence Interval.]*

Qualitative data reinforce the quantitative findings regarding the importance of peer support. One guardian described:

*I saw my son going to his friends’ home to study. Particularly before examinations, he takes help from his intelligent friends. Thus, he completes his examination syllabus and does better in the examination.*

Chi-square analyses confirmed significant associations between academic performance and class level (χ² = 25.47, df = 4, p < .001), difficulty in understanding lessons (χ² = 20.78, df = 1, p < .001), marital status of female students (χ² = 13.26, df = 1, p < .001), family income (χ² = 23.09, df = 1, p < .001), smartphone usage (χ² = 15.47, df = 1, p < .001), bullying (χ² = 37.98, df = 1, p < .001), peer influence (χ² = 26.68, df = 1, p < .001), and teachers’ behavior (χ² = 20.99, df = 1, p < .001) (see Table 4).

**Table 4:** Associations between selected variables and academic performance, school attendance, and absenteeism.

Variables	Academic Performance			School Attendance			Absenteeism		
	χ²	df	*P*-value	χ²	df	*P*-value	χ²	df	*P*-value
Sex	0.678	1	0.410	1.961	1	0.161	1.925	1	0.165
Age	2.065	3	0.559	1.943	3	0.584	1.275	3	0.735
Class	25.47	4	0.000	8.132	4	0.087	21.00	4	0.000
Number of teachers	52.614	2	0.000	0.657	2	0.720	34.06	2	0.000
Difficulty in understanding lessons	20.779	1	0.000	0.371	1	0.543	26.99	1	0.000
Parental education	0.774	2	0.679	7.602	2	0.022	1.171	2	0.557
Marital status of female students	13.255	1	0.000	0.732	1	0.392	4.128	1	0.042
Family income	23.093	1	0.000	2.553	1	0.110	17.25	1	0.000
School distance	0.568	1	0.451	6.414	1	0.011	0.104	1	0.747
Smartphone usage	15.472	1	0.000	0.073	1	0.788	10.50	1	0.001
Bullying	37.975	1	0.000	1.510	1	0.219	21.21	1	0.000
Peer help for study	26.682	1	0.000	4.233	1	0.040	16.53	1	0.000
Teachers’ behavior	20.987	1	0.000	2.380	1	0.123	10.98	1	0.001

#### Challenges in teaching and learning.

Students reported substantial learning difficulties across several subjects. The majority experienced challenges in Mathematics (79.2%) and English (71.0%), followed by General Science (48.7%) and Information and Communication Technology (ICT) (30.3%). Specific learning barriers were also explored. Over half of the students reported difficulty understanding or solving mathematical problems (51.3%), while 49.6% struggled to memorize English vocabulary and 40.3% had difficulty understanding English passages. In addition, 23.5% reported confusion with English grammar, and 54.6% indicated that teachers often teach quickly without clearly explaining concepts, suggesting that both subject complexity and instructional practices contribute to learning challenges.

School and household factors further contributed to these difficulties. Inadequacy of teachers (75.3%), lack of resources in schools (72%), lack of private tutoring or academic support at home (46.2%), and parents’ indifference towards their children#39;s studies (34.4%) are factors thought to contribute to this problem. Consistent with these findings, chi-square analysis indicated a strong association between the number of teachers and academic performance (χ² = 52.61, df = 2, p < .001) (see Table 4). Teachers sometimes pressure students through irrational means, including punishment. One teacher noted:

*Teachers want to motivate their students to learn, even by adopting different strategies. They impose fines on students for their absence from school. Sometimes, teachers beat students without considering the actual causes of absenteeism. These strategies may lead to adverse outcomes, including psychological disruptions, feelings of helplessness, and poor learning.*

Additional insight was found in the following statement of another teacher:

*We often want to help our students, but due to large classes and lack of training, we cannot teach them properly. As a result, many students cannot understand their lessons and perform poorly in examinations.*

These statements by teachers highlight the connection between large class size, teaching inadequacy, and poor student achievement, supporting quantitative evidence that teacher- and school-related factors significantly predict both academic performance and absenteeism. Furthermore, classes in rural areas often do not occur as scheduled and teacher attendance is also very irregular at schools, particularly during the rainy season. These conditions limit students’ opportunities to discuss their learning difficulties with teachers.

#### Early marriage and academic performance.

Female student participation in education remains low in *Haor* areas. Among the female students surveyed, the majority were unmarried (81.5%), of whom nearly half (46.7%) had married before the age of 18. Eve-teasing (78.3%) and early marriage (69.8%) were the most frequently reported factors hindering academic performance. One female teacher stated of eve-teasing:

*Female students often face inappropriate comments, stalking, and offensive gestures while going to and returning from school, causing them significant discomfort and fear.*

Other contributors to low female education include safety concerns during travel to school (43.4%), lack of parental support for girls’ education (41.3%), economic hardship (39.7%), long travel distance (36.5%), lack of separate facilities for girls at schools (28.6%), irregular attendance due to family obligations (21.7%), household responsibilities (17.5%), and parental pressure for early marriage (7.4%). Early-married students had poorer academic performance (85.7%) and higher chronic absenteeism (45.7%) than unmarried peers (16.8%). Chi-square tests confirmed significant associations between the marital status of female students and academic performance (χ² = 13.255, df = 1, p < .001) (see Table 4).

Qualitative interviews support quantitative findings on the associations between early marriage, absenteeism, and academic performance. One guardian shared that:

*Girls in my neighborhood are often taken out of school after puberty. Parents say education is useless for them; they are taught at home to help them prepare for their future married life.*

### School attendance and absenteeism

School attendance and student absenteeism were influenced by several factors. Quantitative data showed that male students were more likely to attend school regularly (52.8%) than females, whereas chronic absenteeism was higher among females (43.2%) than males (33.3%). Age also played a role: students aged 13–14 years attended more regularly (58.8%) than those aged 17–18 years (52.4%), while chronic absenteeism increased with age (38.1%). Class level further differentiated attendance patterns, with Class Seven students showing the highest regular attendance (63.9%) and lowest chronic absenteeism (11.1%), whereas Class Nine students had the poorest attendance (37.5%) and the highest chronic absenteeism (57.5%) (see Table 2).

According to data, irregular attendance was primarily influenced by economic hardship (92.6%), marriage during study (63.5%), household work (58.2%), fear of harsh teachers (55.6%), and school distance (42.3%). Environmental and logistical barriers, including waterway obstacles (21.2%) and muddy roads (39.7%), also restricted attendance. Qualitative data revealed that fear of difficult subjects and the threat of punishment also discouraged attendance. One teacher explained:

*Weak students are often intimidated and remain inattentive during lectures on English and Mathematics. They cannot answer while asking questions about the discussed lessons. If we punish them for their failure to respond, they are likely to miss the next class.*

Another key informant highlighted the pressure between schooling and economic responsibilities:

*I know one student who consistently performs well, but often misses school on market days to manage a small shop. His family relies on his income, compelling him to balance his educational aspirations with his household#39;s economic needs.*

These short-term barriers to school attendance often contribute to chronic absenteeism when compounded by structural and socio-cultural challenges. Chronic absenteeism reflected similar obstacles. Economic hardship (95.2%) and waterway barriers (84.7%) were the most prominent, followed by domestic responsibilities (68.8%), income-earning activities (63.5%), and school distance (37.0%). Structural and socio-cultural factors, including marriage during study (23.8%) and flooding (21.2%), as well as poor transport access (39.7%) and lack of transport money (15.9%), further exacerbated absenteeism (see Table 5). Female students faced additional barriers. Safety concerns during travel, including bullying, eve-teasing, and early marriage, limited attendance, and contributed to poor academic outcomes. One female teacher explained:

**Table 5:** Causes of irregular school attendance and chronic absenteeism (N = 189).

Causes	Attendance n (%)	Absenteeism n (%)
Economic hardship	175 (92.6)	180 (95.2)
Waterway barriers/absence of boats	40 (21.2)	160 (84.7)
Marriage during study/early marriage	120 (63.5)	45 (23.8)
Household work/domestic responsibilities	110 (58.2)	130 (68.8)
Fear of hard/rough teachers	105 (55.6)	110 (58.2)
School distance	80 (42.3)	70 (37.0)
Muddy roads/poor road conditions	75 (39.7)	65 (34.4)
Income-earning activities	50 (26.5)	120 (63.5)
Abrupt flooding	45 (23.8)	40 (21.2)
Poor transport access	—	75 (39.7)
Lack of transport money	—	30 (15.9)
Low parental awareness	—	28 (14.8)
Health issues	—	25 (13.2)
Safety concerns	—	20 (10.6)

**Note.** *Multiple responses were allowed*

*Some girls genuinely aspire to continue their education and perform well academically; however, persistent fear of harassment and verbal abuse while traveling to and from school discourages them. Over time, this fear ultimately leads many to discontinue their studies.*

Chi-square analysis further highlighted patterns across factors affecting attendance and absenteeism. School attendance was significantly associated with parental education (χ² = 7.60, df = 2, p = .022), school distance (χ² = 6.41, df = 1, p = .011), and peer influence (χ² = 4.23, df = 1, p = .040), demonstrating the role of parental support and accessibility in facilitating consistent attendance. In contrast, absenteeism was associated with structural and social challenges, including class level (χ² = 21.00, df = 4, p < .001), number of teachers (χ² = 34.06, df = 2, p < .001), difficulty in understanding lessons (χ² = 26.99, df = 1, p < .001), family income (χ² = 17.25, df = 1, p < .001), bullying (χ² = 21.21, df = 1, p < .001), peer influence (χ² = 16.53, df = 1, p < .001), teachers’ behavior (χ² = 10.98, df = 1, p = .001), and marital status of female students (χ² = 4.13, df = 1, p = .042) (see Table 4).

Logistic regression analyses identified significant predictors. For school attendance, parental education positively predicted regular attendance (B = 0.910, p = .009, OR = 2.48, 95% CI [1.25, 4.93]), whereas school distance (B = –0.819, p = .013, OR = 0.44, 95% CI [0.23, 0.84]) and peer help for study (B = –0.832, p = .037, OR = 0.44, 95% CI [0.20, 0.95]) negatively predicted attendance. Household work showed a marginal positive effect (B = 0.630, p = .054, OR = 1.88, 95% CI [0.99, 3.56]) (see Table 6).

**Table 6:** Logistic regression analysis predicting school attendance.

Variables	School Attendance							
	B	SE	Wald	df	*P*-value	OR (Exp(B))	95% CI for OR	
							Lower	Upper
Doing household work	0.630	0.327	3.715	1	0.054	1.878	0.989	3.563
Income-earning activities	−1.401	0.988	2.010	1	0.156	0.246	0.036	1.708
Parental education	0.910	0.350	6.750	1	0.009	2.484	1.250	4.933
Family income	−0.613	0.330	3.448	1	0.063	0.542	0.283	1.035
School distance	−0.819	0.330	6.149	1	0.013	0.441	0.231	0.842
Smartphone usage	0.612	0.395	2.396	1	0.122	1.843	0.850	3.999
Bullying	0.238	0.342	0.485	1	0.486	1.269	0.649	2.481
Peer help for study	−0.832	0.399	4.358	1	0.037	0.435	0.199	0.950
Teachers’ behavior	−0.466	0.373	1.562	1	0.211	0.627	0.302	1.303
Constant	0.793	0.509	2.426	1	0.119	2.211	—	—

[**Note.** *N = 189.P <.05 values are significant. OR = Odds Ratio; CI = Confidence Interval*.]

For absenteeism, household work reduced the odds of being absent (B = –1.082, p = .022, OR = 0.34, 95% CI [0.14, 0.85]), and family income strongly reduced absenteeism (B = –1.350, p < .001, OR = 0.26, 95% CI [0.13, 0.53]). In contrast, bullying (B = 1.361, p < .001, OR = 3.90, 95% CI [1.90, 7.99]) and teachers’ behavior (B = 1.146, p = .009, OR = 3.15, 95% CI [1.33, 7.43]) increased absenteeism (see Table 7).

**Table 7:** Logistic regression analysis predicting absenteeism.

Variables	Absenteeism							
	B	SE	Wald	df	*P*-value	OR (Exp(B))	95% CI for OR	
							Lower	Upper
Doing household work	–1.08	0.471	5.28	1	.022	0.34	0.14	0.85
Parental education	−0.52	0.375	1.897	1	0.168	0.597	0.286	1.244
Family income	−1.35	0.368	13.489	1	<0.001	0.259	0.126	0.532
School distance	0.013	0.365	0.001	1	0.973	1.013	0.495	2.073
Bullying	1.361	0.366	13.830	1	<0.001	3.900	1.904	7.991
Peer help for study	−0.92	0.371	6.162	1	0.013	0.398	0.193	0.824
Teachers’ behavior	1.146	0.439	6.815	1	0.009	3.145	1.331	7.434
Constant	−0.50	0.528	0.911	1	0.340	0.604	—	—

[**Note.** *N = 189.P <.05 values are significant. OR = Odds Ratio; CI = Confidence Interval*.]

### School initiatives for educational challenges

Schools in the *Haor* region generally implement diverse strategies to enhance learning and attendance. The most frequent initiatives are community meetings and student motivation sessions (79.9%), followed by parents’ meetings (73.0%) and giving special attention to students (61.4%). Punishment is applied in 64.6% of cases, while informing parents occurs in 46%. Less common strategies include motivating parents (31.2%), not permitting to attend the next day’s class (18.5%), awarding attendance prizes (18.0%), and class-wise student meetings (15.9%) (see Table 8). During qualitative interviews, teachers reported that punitive measures are effective in increasing students’ attendance at school. Parental involvement is inconsistent in addressing students’ problematic behaviors. One teacher stated:

**Table 8:** Initiatives taken for reducing schooling problems (N = 189).

Initiatives	Frequency	Percentage
Community meeting	151	79.9
Giving motivations to students	151	79.9
Arranging parents’ meetings	138	73.0
Giving punishment	122	64.6
Giving special attention	116	61.4
Informing parents	87	46.0
Motivating parents of the students	59	31.2
Not permitting to attend the next day’s class	35	18.5
Giving prizes on attendance	34	18.0
Class-wise meeting with students	30	15.9

**Note.** *Multiple responses were allowed*

*Even though our school holds meetings for students, most parents do not attend. Children continue to skip classes without valid reasons.*

Additional perspective was shared by one head teacher:

*I discuss students#39; irregularity and absenteeism in our meetings, but this topic is not included in our regular meetings. Without continuous effort and consistent follow-up, these initiatives would not make much difference for the students.*

This emphasizes why current interventions have limited effect, complementing quantitative patterns of irregular attendance, absenteeism, and poor academic outcomes.

### Required initiatives for educational improvement

#### Public awareness campaigns.

Respondents highlighted that current school initiatives are insufficient to improve educational outcomes. Many children lack aspirations, and parental detachment further reduces engagement. To improve this situation, respondents suggested public awareness campaigns targeting both students and parents. As one teacher recommended:

*In Haor areas, guardians are generally unaware of the importance of their children’s education. They prioritize income generation over education. Therefore, a comprehensive public awareness campaign is essential to raise awareness among students and their parents. This initiative can be effectively implemented through yard meetings and community gatherings. Local institutions such as mosques and community organizations can be engaged to implement the awareness campaign.*

This statement highlights the need for multi-level awareness interventions to promote education in *Haor* areas.

#### Financial support for students.

Poverty remains a significant barrier to education, particularly for boys who are often engaged in income-generating activities. Respondents recommended increasing educational stipends for financial support:

*Haor areas are poverty-stricken areas. Large families seek to increase their income by having their male children engage in income-generating activities. Hence, growing stipends for male students would encourage parents to prioritize their boys’ education.*

Additionally, in families where boys become primary earners due to the absence of earning members, targeted financial support is essential to enable continued schooling.

#### Counseling and motivational support.

Some guardians hold beliefs that children must contribute to household work to understand life realities, hindering educational motivation. Teachers suggested counseling interventions to address these attitudes:

*Community and family counseling services can play a crucial role in addressing attitudinal ambivalence among guardians. Through these initiatives, both children and their guardians can be made to understand the long-term value of education.*

Another teacher emphasized to specifically motivating students:

*Haor children often lack the ambition to complete their studies. They only think about their families#39; day-to-day survival. So, it is essential to encourage them to think beyond this mindset. Counseling services at regular intervals can introduce motivation and commitment to education among these students.*

In addition, home visits, community awareness campaigns, and school-based guardian programs were recommended to shift parental priorities from short-term financial gain to education.

#### Promoting female education.

Female education faces unique barriers, including menstruation, early marriage, transportation challenges, and socio-cultural norms. Respondents highlighted the need for gender-sensitive interventions. One female teacher noted:

*Many of our female students miss school during their menstrual periods because they lack access to proper sanitary materials and private facilities at school. This not only affects their attendance but also lowers their achievements.*

Safety and community support were emphasized by another teacher:

*A dedicated committee should be established to ensure the safety and well-being of female students. This committee would be responsible for raising parental awareness and developing gender-sensitive school safety plans. This committee will implement preventive initiatives, including regular awareness sessions, community engagement programs, and early risk identification.*

The female teacher further added:

*Appointing dedicated staffs with expertise in child protection, gender issues, and psychosocial support is essential in Haor schools to effectively address gender-based issues.*

#### Addressing the school infrastructure challenge.

Overcrowded classrooms were observed in several schools due to a shortage of classrooms and a small area of existing classrooms, limiting teachers’ capacity to engage all students effectively during lecture delivery, as one teacher highlighted:

*Due to overcrowded classrooms, students sitting at the back are unable to listen to the lecture uninterrupted and participate in classroom discussions. I cannot provide individual attention to all students. Improving infrastructure is crucial to enhancing learning environments.*

## Discussion

This study, conducted in the *Haor* area of Sunamganj District in Bangladesh, explores the educational challenges and current and potential initiatives to enhance educational performance among secondary school students. The findings reveal a complex interplay among socio-demographic and institutional factors that influences students’ academic performance, school attendance, and absenteeism. Rather than operating in isolation, these factors interact in a layered manner, where structural disadvantage amplifies individual and school-level vulnerabilities. Academic performance and absenteeism are influenced by a broader range of factors than school attendance, and both are significantly associated with class level, number of teachers, learning difficulties, family income, smartphone use, bullying, peer influence, and teachers’ behavior. In contrast, attendance is primarily shaped by parental education, school distance, and peer influence, indicating fewer but more context-specific determinants. Notably, peer influence emerges as a common factor across all three outcomes. This pattern suggests that educational inequality in *Haor* areas is not merely a pedagogical issue but a reflection of broader social realities.

Our findings regarding academic performance indicate that family income is the most significant positive predictor of success. Students from well-off families are more than eight times as likely to achieve good academic outcomes as those from economically disadvantaged families. The influence of family income on student performance is consistent with the findings of Guo et al. [19], who found that higher family income enhances academic achievement by increasing parental involvement and raising expectations. However, compared to urban or semi-urban contexts, the magnitude of this effect appears more pronounced in *Haor* regions, where alternative support systems (e.g., tutoring centers, digital learning access) are limited. This influence is not merely associative but reflects deep realities embedded within society. This structural reality is explained by Wiborg and Grätz [20] through the concept of “compensatory advantage.” They refer to this concept as the strategy through which affluent families support their children, providing more financial investment, including private tutors, enriched home environments, and learning resources, to compensate for potential academic weaknesses.

Likewise, peer help in study emerged as a significant predictor of academic performance in *Haor* areas. Existing literature has reported statistically significant effects of peer-assisted learning on educational outcomes [21]. This learning process enhances students’ confidence and comprehension, particularly when they resolve complex academic problems through teamwork [22]. However, the dual nature of peer support can complicate this narrative. While it can enhance learning, it may also reinforce exclusion, particularly in socially stratified rural contexts where peer groups tend to form among students from similar socioeconomic backgrounds [23]. Contrary to previous findings, bullying by people on the street and punitive teacher behavior were found to significantly reduce the likelihood of good academic performance in *Haor* contexts. In comparison to other contexts, where bullying is often school-based, the prominence of community-level bullying in *Haor* areas underscores the role of unsafe public spaces in shaping educational outcomes. Samara et al. [24] stated that community bullying undermines students’ psychological well-being, school engagement, and sense of belonging. This adverse stressor ultimately leads students to lower achievement in core subjects, such as Mathematics and Science [25]. Within *Haor* schools, punitive disciplinary practices are prevalent, resulting in reduced students’ educational engagement. Sorensen et al. [26] found that discretionary principal discipline is detrimental to long-term educational outcomes.

Parental education emerged as the single most significant positive predictor of school attendance in *Haor* areas, emphasizing the crucial role of educated parents in maintaining school regularity. The existing literature suggests that more educated parents are more aware of factors that enhance children’s schooling [27]. However, in the *Haor* context, parental education appears to function not only as a cognitive resource but also as a protective factor against environmental and social disruptions. Previous studies suggest variation in the roles of maternal and paternal education [28], while others highlight that parental education alone is insufficient without economic resources [29]. This study reinforces the argument that educational capital and economic capital are mutually reinforcing rather than independent determinants of school participation.

Moreover, our study found school distance as a significant negative predictor of regular attendance, with students living farther from school being significantly less likely to attend regularly. This finding aligns with evidence indicating that travel distance is a significant barrier to active commuting, often exacerbated by lower socioeconomic status and maternal concerns about safety and convenience [30]. Beyond school distance, residential mobility is also an important factor that negatively influences school attendance. In urban contexts, even when students remain in the same school, changing residences can disrupt attendance patterns due to instability and the loss of social networks [31]. In contrast to urban settings, where distance is often a logistical inconvenience, in *Haor* areas, it represents a compounded barrier involving unsafe routes, seasonal flooding, and limited transportation infrastructure. This distinction highlights environmental risk and accessibility constraints in *Haor* areas.

Our study identified several significant predictors of absenteeism in the *Haor* region. Bullying by people on the street emerged as statistically significant and the strongest factor associated with chronic absenteeism. Students who experience such bullying are around four times more likely to be chronically absent from school. This alarming trend reflects a broader pattern observed in other similar contexts, where public spaces often lack safety for children, particularly those walking to school or using unsafe transportation routes [32]. This situation is difficult in rural areas where anti-bullying programs, mental health support, and school counseling services are rare [33]. Inadequate family income also significantly predicts chronic absenteeism in *Haor* schools. Students from low-income households face structural disadvantages, such as food insecurity, poor health, and unstable living conditions, which disrupt consistent school participation [34].

Additionally, economic pressures may compel children to engage in informal labor or take on household responsibilities, thereby increasing the likelihood of chronic absenteeism. In rural areas, poor socioeconomic status, including low parental education, unemployment, and inadequate household income, compounds the likelihood of absenteeism [35]. Punitive teacher behavior, which discourages student engagement, appears as a significant predictor of absenteeism. This aligns with Bacon and Kearney [36], who underscore the influence of teacher behavior on student outcomes and identify school climate and student–teacher relationships as key determinants of absenteeism severity. These findings suggest that absenteeism is not simply an individual choice but a cumulative outcome of adverse school climates, unsafe environments, and economic hardship in *Haor* areas.

This study produced unexpected findings: students who engage in household work are approximately three times more likely to achieve good academic performance than their peers who do not take on such responsibilities. This challenges dominant assumptions in educational research that household labor uniformly hinders academic success. Additionally, these students are also very unlikely to be chronically absent from school. Although household responsibilities may hinder the academic progress of *Haor* students, they can simultaneously cultivate crucial life skills, such as time management and responsibility, and instill aspirations for success and a future free of physically demanding labor. These skills, along with the dream of success, may enable students to maintain a balance between familial responsibilities and academic studies.

While schools undertake initiatives such as community meetings and parental engagement, their impact remains limited. This raises critical questions about the sustainability of current interventions, particularly in geographically and economically constrained settings. Hence, key informants emphasized the need for more collaborative and context-specific strategies. Keller and Grumbach [37] highlight the importance of coordinated efforts among stakeholders. Compared with generic educational interventions, *Haor*-specific strategies, such as transport support during flooding seasons or localized safety measures, are likely to yield more meaningful outcomes. Establishing school-level committees staffed by trained child protection professionals was recommended to address bullying and safety concerns. Community, family, and individual counseling services were also suggested, aligning with Watson et al. [38]. These recommendations reflect a shift from reactive to preventive approaches, emphasizing early intervention and holistic support systems. Additional strategies, including awareness campaigns, community engagement, and financial assistance, were identified as essential. Such interventions highlight the importance of integrating social protection measures with educational policies to address structural inequalities.

This study provides empirical evidence on how socio-economic, institutional, and environmental factors shape educational engagement among secondary-level students in a low-income, under-resourced context in Bangladesh. Rather than attributing poor educational outcomes only to students’ academic limitations, this research emphasizes socio-demographic, behavioral, and institutional dimensions that also constrain students’ opportunities and participation. By offering insights from the *Haor* context, where educational trajectories are deeply influenced by chronic poverty, geographic isolation, and traditional sociocultural norms, this study would contribute to educational development in vulnerable areas of the Global South, including *Haor*, coastal, and riverine areas.

However, as this study was conducted in selected *Haor* areas of Bangladesh, it may not fully represent the diversity of educational experiences across other marginalized or flood-prone regions of the country. The study design also restricts the ability to observe long-term changes in academic performance in these areas. In addition, reliance on students’ self-reported data may not accurately reflect the realities, particularly regarding school practices. The identification of inattentive students relied on teachers’ subjective judgment, which may introduce bias. Furthermore, although the independent variables might operate at multiple levels (student, teacher, and school), the analysis was done to produce an overall picture. Multilevel modeling was not applied, as the primary aim was exploratory and descriptive. Since the study was conducted in a limited number of schools and with a relatively small sample size, it was not feasible to establish hierarchical or causal relationships between variables.

Future research should adopt longitudinal designs to track changes in academic outcomes and attendance. Experimental or evaluative studies may be more valuable to explore the effectiveness of school interventions. Comparative studies across multiple wetland or flood-prone areas could provide a more comprehensive understanding of these aspects, accounting for contextual variations in policy interventions. Future studies aiming to quantify school-level or teacher-level effects should conduct multilevel analysis to account for nested dependencies.

## Conclusion

This study examined the factors influencing students’ educational performance, irregularity, and absenteeism in the *Haor* region of Bangladesh. The findings reveal that children’s academic outcomes are significantly shaped by family income, exposure to street-level bullying, teacher behavior, and access to peer support. Students from economically well-off households and those having supportive peers perform better, while those facing bullying and strict disciplinary practices are vulnerable to poor educational outcomes. Furthermore, school attendance is significantly influenced by structural and intergenerational barriers, including school distance and parental education. Family and community-level factors, including bullying on the street and financial instability, impact absenteeism. These insights reveal a web of interrelated personal, social, and institutional factors lowering the status of *Haor* education. Although schools have been taking several initiatives to address these issues, a systemic gap persists that requires urgent attention to ensure comprehensive action.

Based on the study findings, several policy recommendations emerge to address the educational challenges in the *Haor* region. Context-specific anti-bullying strategies should be developed, such as establishing school-level committees with local community authority figures to ensure safe walking to and from schools for students, and providing school transport funded through contributions from local philanthropists, affluent individuals, and government support to reduce irregular attendance. Teacher training programs should be initiated with a focus on student-centered pedagogy. Schools can institutionalize collaborative learning to strengthen academic resilience and encourage peer mentoring to improve the performance of slow learners. To address economic barriers, targeted social protection schemes should be implemented. The government may provide stipends to all students and increase the stipend amount for students from vulnerable families.

Additionally, punitive disciplinary measures should be replaced with inspiring approaches that recognize regular attendance and academic progress. Finally, promoting parental involvement through parent–teacher meetings and community counseling can foster shared responsibility for children’s education. These recommendations align with school social work frameworks and call for an integrated, inclusive, and contextually grounded approach to improving education in *Haor* areas.

## Supporting information

S1 File (PDF)
