# Supplementary material for: Education in Haor areas of Bangladesh: A mixed-methods study on academic performance, school attendance, and school intervention
Source: PLoS One. 2026 May 11;21(5):e0348890. doi: 10.1371/journal.pone.0348890 (PMC13160321; doi:10.1371/journal.pone.0348890)
Supplement: S1 File — (PDF) [file pone.0348890.s001.pdf]

Table 1: Descriptive statistics of key variables (n = 189)

Variables	Category	Frequency	Percentage
Doing household work	No	95	50.3
	Yes	94	49.7
Doing income-earning activities	No	183	96.8
	Yes	6	3.2
Parental education	Below secondary	124	65.6
	Secondary	43	22.8
	Above secondary	22	11.6
Family income	Adequate	99	52.4
	Inadequate	90	47.6
School distance	No	104	55.0
	Yes	85	45.0
Smartphone usage	No	61	32.3
	Yes	128	67.7
Bullying	No	107	56.6
	Yes	82	43.4
Peer influence	No	62	32.8
	Yes	127	67.2
Teachers' behavior	Punitive	53	28.0
	Supportive	136	72.0
Difficulty in understanding lessons	No	70	37.0
	Yes	119	63.0
Academic performance	Poor	57	30.2
	Good	132	69.8
School attendance	Irregular	81	42.9
	Regular	108	57.1
Absenteeism	Occasional	118	62.4
	Chronic	71	37.6

Table 2: Marital status and early marriage

Variables	Category	Frequency	Percentage
Married status of female students (n = 81)	No	66	81.5
	Yes	15	18.5
Marriage before 18 (n = 15)	No	8	53.3
	Yes	7	46.7

Table 3. Factors hindering academic performance among female students (n = 189)

Factors	Frequency	Percentage
Eve-teasing	148	78.3
Early marriage	132	69.8
Lack of parental support for girls' education	78	41.3
Economic hardship of the family	75	39.7
Safety concerns during travel to school	82	43.4
Long distance between home and school	69	36.5
Household responsibilities	33	17.5
Irregular attendance due to family obligations	41	21.7
Lack of separate facilities for girls at schools	54	28.6
Parental pressure for early marriage	14	7.4

Note. *Multiple responses were allowed*

Table 4: Students' responses after teachers' reproaching (n = 102)

Responses	Frequency	Percentage
Feel very sad	43	42.2
Feel crying	31	30.4
Feel frightened	43	42.2
Disinterest go to school next day	51	50.0
Sometimes feel nothing	27	26.5

Note. *Multiple responses were allowed*

Table 5: Learning difficulties in subjects (n=119)

Subjects	Frequency	Percentage
Mathematics	94	79.2
English	84	71.0
Science	58	48.7
Information and Communication Technology (ICT)	36	30.3

Note. *Multiple responses were allowed*

Table 6: **Causes of learning difficulties (n=119)**

Causes	Frequency	Percentage
Do not understand and solve math problems	61	51.3
Students struggle to understand English passages	48	40.3
Difficulty in memorizing English words	59	49.6
Confusion with English grammar	28	23.5
Teachers often teach quickly without explaining concepts	65	54.6

Note. *Multiple responses were allowed*

Table 7. Factors contributing to learning difficulties (n = 119)

Contributing factors	Frequency	Percentage
Inadequacy of teachers	90	75.3
Lack of resources in schools	86	72.0
Parents' indifference toward children's studies	41	34.4
Lack of private tutoring or academic support at home	55	46.2

Note. *Multiple responses were allowed*

Table 8: Types of physical punishment experienced by students (N = 189)

Types	Frequency	Percentage
Hitting with objects (sticks, scales, books, pens, dusters)	170	90.0
Twisting ears	126	66.7
Public humiliation (verbal abuse, name-calling, shaming)	117	62.0
Forced standing for long periods	110	58.0
Kneeling or stress positions	87	46.0
Pulling ears with hands under thighs	82	43.3
Extra physical tasks (cleaning, carrying loads)	77	41.0
Stretching hair or other body parts	55	29.2

Note. *Multiple responses were allowed*

Table 9: Reasons for physical punishment (N = 189)

Reasons	Frequency	Percentage
Irregular attendance	164	86.8
Non-compliance with teacher instructions	119	63.0
Late arrival to school	115	61.0
Inattentiveness in class	112	59.3
Poor academic performance	108	57.0
Fleeing from school	102	53.8
Failure to complete homework	94	49.5
Lack of school materials (books, pens, uniform)	83	44.0

Note. *Multiple responses were allowed*

Table 10: Body parts where punishment given (n = 150)

Body parts	Frequency	Percentage
Hands	108	73.0
Legs	68	45.9
Back	80	54.1
Head	50	33.8
Cheeks	54	36.5
Ears	52	35.1
Randomly	5	3.4

Note. *Multiple responses were allowed*

Table 11: Causes of early marriage (n=189)

Causes	Frequency	Percentage
Early marriage is considered a tradition	135	71.4
Poverty	134	70.9
Parents see marriage as the only option for the future	124	65.6
Insecurity and harassment	115	60.8
Fear of social stigma	40	21.2
Limited awareness of legal age of marriage	35	18.5
Weak enforcement of child marriage laws	32	16.9

Note. *Multiple responses were allowed*

Table 12: Causes for not asking questions in class (n=88)

Causes	Frequency	Percentage
Feel shy that others will laugh	51	58.0
Teacher might get angry	55	62.5
Do not understand the lesson well	59	67.0
Stay quiet because asking questions may interrupt the class	58	65.9
Lack of confidence due to weak academic background	28	31.8
Feeling that teachers do not value students' questions	34	38.6

Note. *Multiple responses were allowed*

Table 13. Events per variable (EPV) for logistic regression models

Models	Outcome category	Number of events	Number of predictors	EPV	Interpretation
Academic performance	Good (coded as 1)	132	9	14.67	Adequate
School attendance	Regular (coded as 1)	108	9	12.00	Adequate
Absenteeism	Chronic (coded as 1)	71	7	10.14	Adequate

Table 14. Model fit statistics for academic performance, school attendance, and absenteeism

Models	−2 Log likelihood	Cox & Snell r^2	Nagelkerke r^2	Hosmer–Lemeshow χ^2	P-value
Academic performance	121.540	0.441	0.624	5.597	0.692
School attendance	227.628	0.149	0.200	11.514	0.174
Absenteeism	193.141	0.261	0.355	10.649	0.222
